# Fast Swept-Wavelength, Low Threshold-Current, Continuous-Wave External Cavity Quantum Cascade Laser

**DOI:** 10.1186/s11671-018-2765-1

**Published:** 2018-10-26

**Authors:** Xuefeng Jia, Lijun Wang, Zhiwei Jia, Ning Zhuo, Jinchuan Zhang, Shenqiang Zhai, Junqi Liu, Shuman Liu, Fengqi Liu, Zhanguo Wang

**Affiliations:** 1 Key Laboratory of Semiconductor Materials Science & Beijing Key Laboratory of Low Dimensional Semiconductor Materials and Devices, Institute of Semiconductors, Chinese Academy of Sciences, Beijing, 100083 China; 2 Center of Materials Science and Optoelectronics Engineering, University of Chinese Academy of Sciences, Beijing, 100049 China

**Keywords:** Quantum cascade laser, External cavity, Fast swept-wavelength

## Abstract

We present a low threshold-current and fast wavelength-tuning external cavity quantum cascade laser (EC-QCL) using a scanning galvanometer in the Littman-Metcalf cavity geometry. The EC-QCL could repeatedly swept at 100 Hz over its full tuning range of about 290 nm (2105 cm^−1^ to 2240 cm^−1^), providing a scan rate of 59.3 μm s^−1^. The continuous-wave (CW) threshold current of the EC-QCL was as low as 250 mA and the maximum output power was 20.8 mW at 400 mA for a 3-mm-long QCL gain chip. With a sawtooth wave modulation, a scan resolution of < 0.2 cm^−1^ can be achieved within the tuning range. The low power-consumption and fast swept-wavelength EC-QCL will be beneficial to many applications.

## Background

The mid-infrared (MIR) region of the electromagnetic spectrum is the molecular fingerprint region, since fundamental ro-vibrational transition energies of most molecules lie in this spectral region. Laser absorption spectroscopy in the MIR region is important for a diverse number of applications such as medical breath analysis, atmospheric pollutants sensing, and industrial effluents monitoring [1–3]. Particularly, with the fast development of MIR lasers, the performance of optical instruments based on spectroscopy method has been greatly improved to provide rapid, sensitive, and accurate measurements.

For the laser absorption spectroscopy, a tunable single-frequency laser with narrow linewidth and modest power is required. Distributed feedback (DFB) quantum cascade lasers (QCLs) [1] are suitable light sources for these applications because of their very narrow linewidth [4], high output power, and room-temperature continuous-wave (CW) operation. However, a single DFB laser has a very limited tuning range of a few cm^−1^ (~ 10 cm^−1^) via slow temperature tuning, which limits its usefulness for broadband absorption features and multi-species gas detection [5]. DFB arrays have achieved an impressive tunability over 220 cm^−1^. However, DFB arrays need electron beam lithography to fabricate different grating periods, which is complex and expensive. Moreover, DFB arrays need beam combining of different wavelengths for sensing applications [6, 7].

External cavity quantum cascade lasers (EC-QCLs) are widely used as reliable, broadly tunable light sources, which can provide a tuning range greater than 300 cm^−1^ [8] with slow scan by stepper motor. For traditional EC-QCL, the mode-hop free tuning can be achieved by mode tracking system which was proposed by Wysocki et al. [9]. The laser current and the EC length are modulated with phase-matched triangular voltage ramps during the tuning process. However, this only allows mode-hop-free tuning of ~ 1 cm^−1^ at any wavelength inside the full tuning range of the EC-QCL [10]. A high wavelength tuning rate EC-QCL is needed for reducing the measurements time of chemical mixtures in the gas phase. Rapidly swept EC-QCLs have been designed with intra-cavity micro-eletromechanical system (MEMS) or acousto-optic modulator, which can sweep > 100 cm^−1^ on a sub-ms timescale [11]. Unfortunately, these rapidly swept EC-QCL systems have low spectral resolutions around ~ 1 cm^−1^, which is not sufficient for the narrow absorption features.

Recently, a swept-wavelength EC-QCL source for measurements of broad absorption features was developed by M.C. Phillips et al. [12, 13]. The swept-wavelength EC-QCL can be tuned more than 100 cm^−1^ at a sweep rate of 200 Hz with an average output power of 11 mW at the peak of the tuning curve at 50% duty cycle. However, pulsed operation would introduce line-broadening due to the chirped current. In this paper, we use the scanning galvanometer in the Littman-Metcalf cavity geometry to realize a fast swept-wavelength EC-QCL with a tuning range of 135 cm^−1^ from 2105 to 2240 cm^−1^ (4.46–4.75 μm). The threshold current was as low as 250 mA in CW operation at room temperature. Time-resolved measurement using the step-scan Fourier transform infrared (FTIR) technique was performed for the EC-QCL repeatedly swept at 100 Hz. Laser spectrum analyzer was used to evaluate the spectral resolution. With a sawtooth wave modulation, a spectral resolution of < 0.2 cm^−1^ can be achieved within the tuning range.

## Methods

**Fig. 1:**
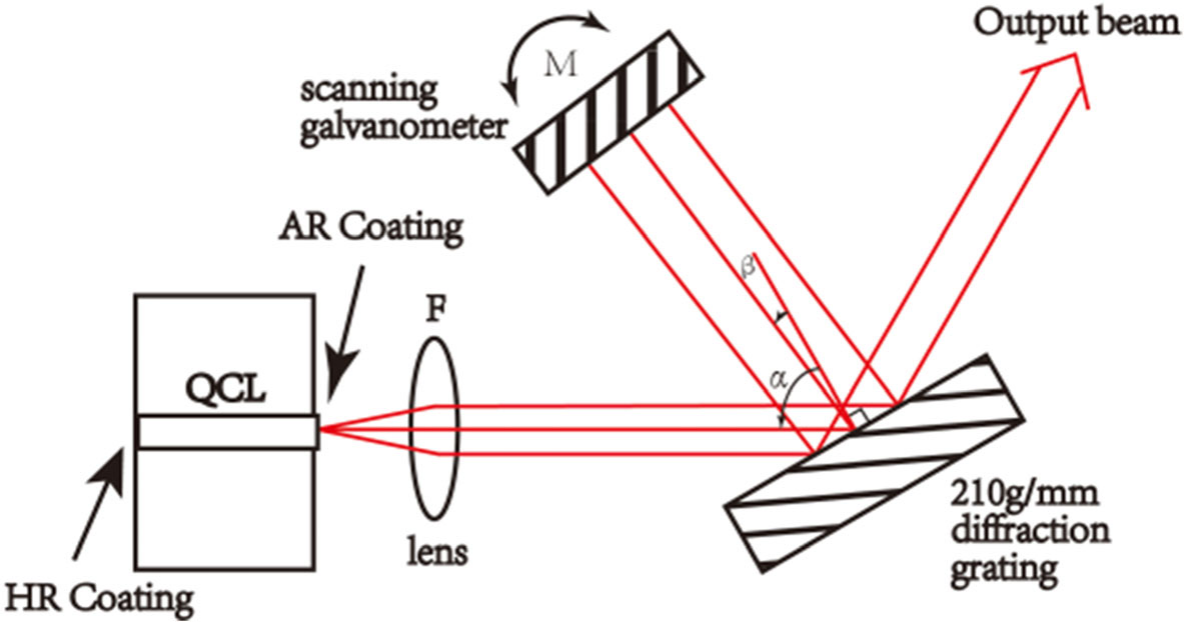
Schematic of Littman-Metcalf external cavity configuration

The EC system is based on the Littman-Metcalf configuration, and consists of three main elements, the gain element, in our case the Fabry–Perot (FP) QCL chip with a collimating lens, a diffraction grating, and a scanning galvanometer, as shown in Fig. 1. The strain-compensated QCL active core comprises 30 periods with In_0.67_Ga_0.33_As/In_0.36_Al_0.64_As as quantum wells and barriers, respectively, similar to that described in [14]. The devices were processed in a buried heterostructure configuration using metal-organic chemical vapor deposition (MOCVD) for the selective regrowth of Fe-doped InP. The FP–QCL gain chip with a ridge width of 12 μm and length of 3 mm was used to construct the EC-QCL. High-reflectivity (HR) coating consisting of Al_2_O_3_/Ti/Au/Ti/Al_2_O_3_ (200/10/100/10/120 nm) and anti-reflection (AR) coating of Al_2_O_3_/Ge (448/35 nm) were evaporated on the rear facet and the front facet of the gain chip, respectively. The FP–QCL chip was mounted epilayer side down on a SiC heat sink with indium solder, wire bonded, then mounted on a holder containing a thermistor combined with a thermoelectric cooler (TEC) to monitor and adjust the heat sink temperature.

The Littman configuration we used consists of a collimating lens with the focal length of 6 mm, a diffraction grating with 210 grooves/mm, and a scanning galvanometer (Thorlabs, GVS111). In the Littman configuration as shown in Fig. 1, first-order light is diffracted into the scanning galvanometer then reflected back into the FP–QCL chip by the diffraction grating and the emitted single-mode laser light is extracted through zeroth-order reflection from the diffraction grating.

The emitted optical power and spectrum from the EC-QCL were measured with a calibrated thermopile detector and a FTIR spectrometer, respectively. All measurements were taken with the FP–QCL chip being held at 25 °C under cw operation.

## Results and Discussion

**Fig. 2:**
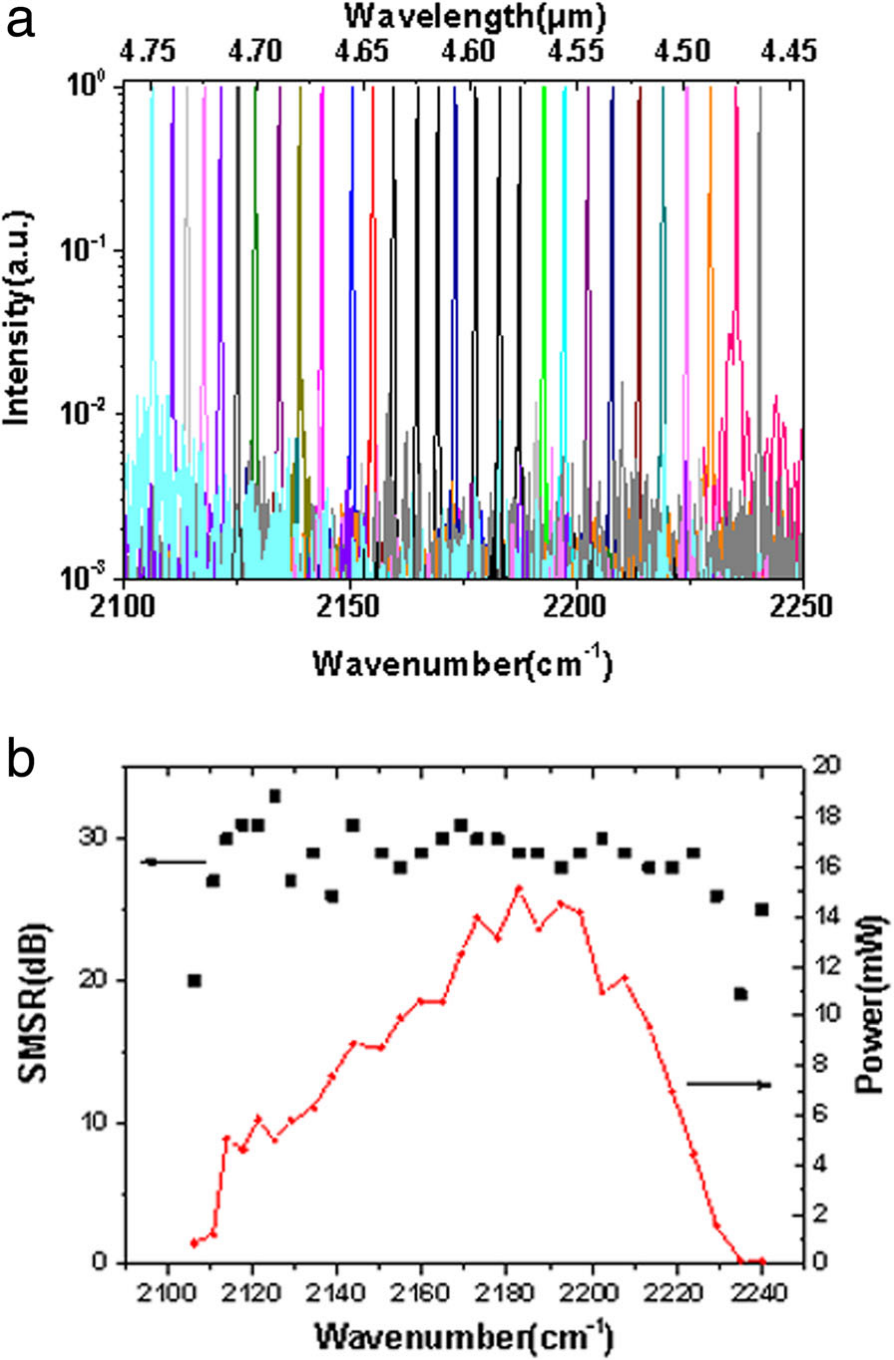
**a** The normalized emission spectra of the EC-QCL measured at 25 °C in cw operation with the current of 330 mA. The adjacent spectrum was measured with the galvanometer rotating step of 0.1°. **b** Measured output power (red curve) and SMSR (black point) of the EC-QCL at different scanning galvanometer angles

**Fig. 3:**
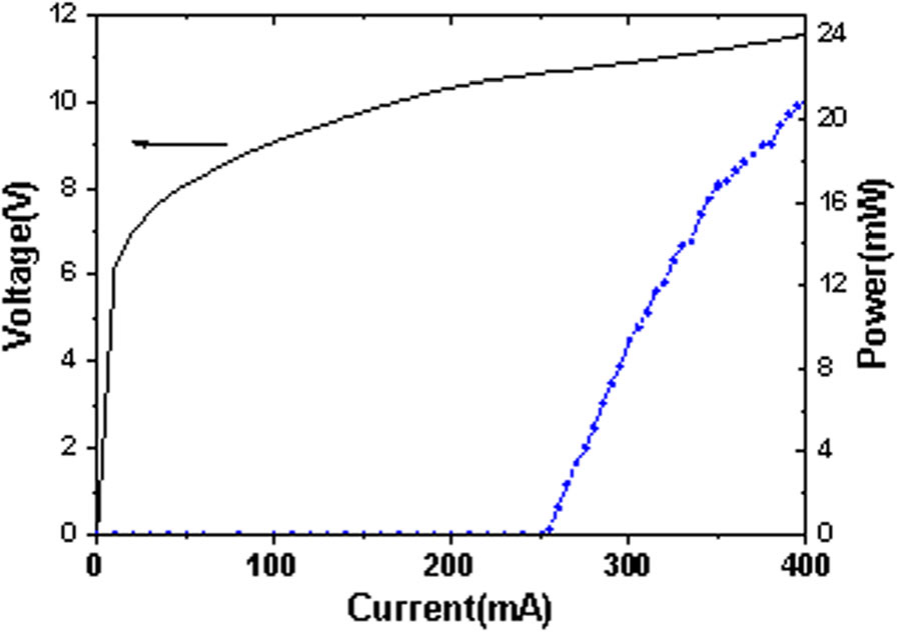
The P-I-V characteristics of the EC-QCL in the central region at 2180 cm^−1^

Figure 2a shows the measured cw spectra at different scanning galvanometer angles with the injection current of 330 mA. The emission peak shifts from 2105 to 2240 cm^−1^ by rotating the galvanometer with the step of 0.1°. Figure 2b shows the measured output power and the side-mode-suppression-ratio (SMSR) at different scanning galvanometer angles same as that in Fig. 2a. A SMSR above 25 dB was realized in almost the whole tuning range. The average output power was about 8 mW and the output power profile was consistent with the electroluminescence spectrum. Figure 3 depicts the power-current-voltage (P-I-V) curves measured for the EC-QCL in the central region at 2180 cm^−1^. The threshold current of the EC-QCL was 250 mA, corresponding to a threshold current density (*J*_th_) of 0.833 kA/cm^2^. The maximum cw output power of 20.8 mW was obtained at 400 mA.

### EC-QCL Scan Characterization

**Fig. 4:**
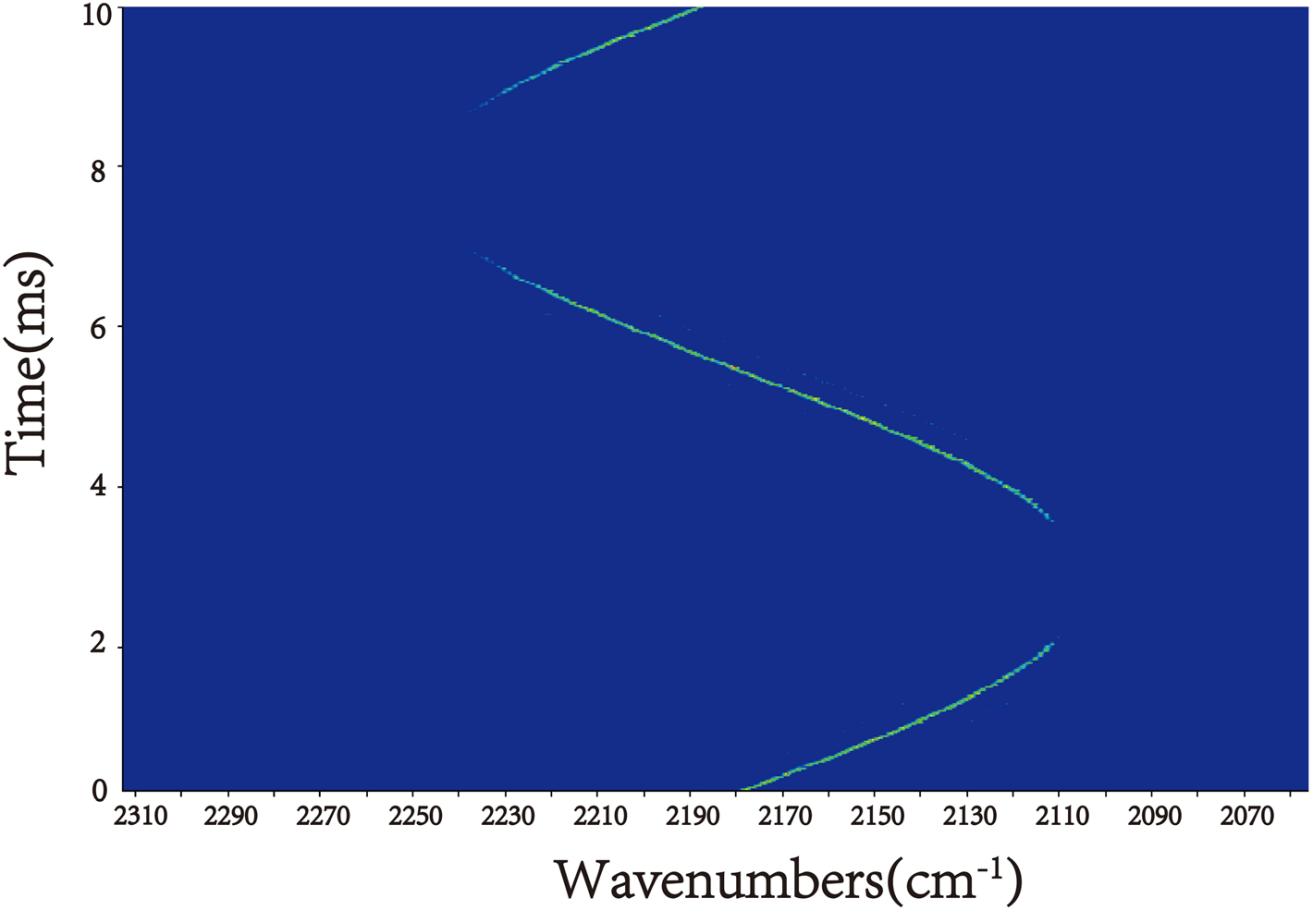
The time-resolved emission peaks of the EC-QCL operated in cw mode at 330 mA and the scanning galvanometer modulation at 100 Hz

We utilized a signal generator to generate a 100 Hz sinusoidal wave. By implementing the sinusoidal wave on the scanning galvanometer, the EC-QCL wavelength can be swept repeatedly in cw mode with the current of 330 mA. The sinusoidal wave amplitude is 3 V which corresponds to the total tuning angle of 3°. For a demonstration of the EC-QCL scan characterization, time-resolved measurement using the step-scan FTIR technique can be applied. This technique was often used to study repeatedly occurred processes [15]. We make the generated signal synchronized with the FTIR, and the measurements were performed with a spectral resolution of 0.2 cm^−1^ and 20 ns time resolution. The time-resolved emission peaks were plotted in Fig. 4. The EC-QCL started at 2180 cm^−1^ then tuning toward lower wavenumbers. After 1/4 periods, the emission peak reached the minimum wavenumber. The wavenumber tuned from 2105 to 2240 cm^−1^ in the next half periods. For the Littman configuration:

1 $$ \uplambda =d/{m}^{\ast}\left(\mathit{\sin}\upalpha +\mathit{\sin}\upbeta \right) $$

where λ is the EC-QCL wavelength, *d* is the grating period, *m* is the diffraction order, and α and β are the angles shown in Fig. 1. The first-order light is reflected to the scanning galvanometer then reflected back into the FP–QCL chip. When the scanning galvanometer rotates an angle of θ, the above formula turns to:

2 $$ \frac{\mathrm{d}\uplambda}{\mathrm{d}\mathrm{t}}={\mathrm{d}}^{\ast}\cos \left(\upbeta +\uptheta \right)\ast \frac{\mathrm{d}\uptheta}{\mathrm{d}\mathrm{t}} $$

In our configuration, *m* = 1, β = 7.7°, *d* = 4.76 μm, and the EC-QCL can operate in a fast-scan mode with the scanning galvanometer swept at 100 Hz with the rate of 12.6 rad/s, providing a wavelength tuning rate of 59.3 μm s^−1^.

**Fig. 5:**
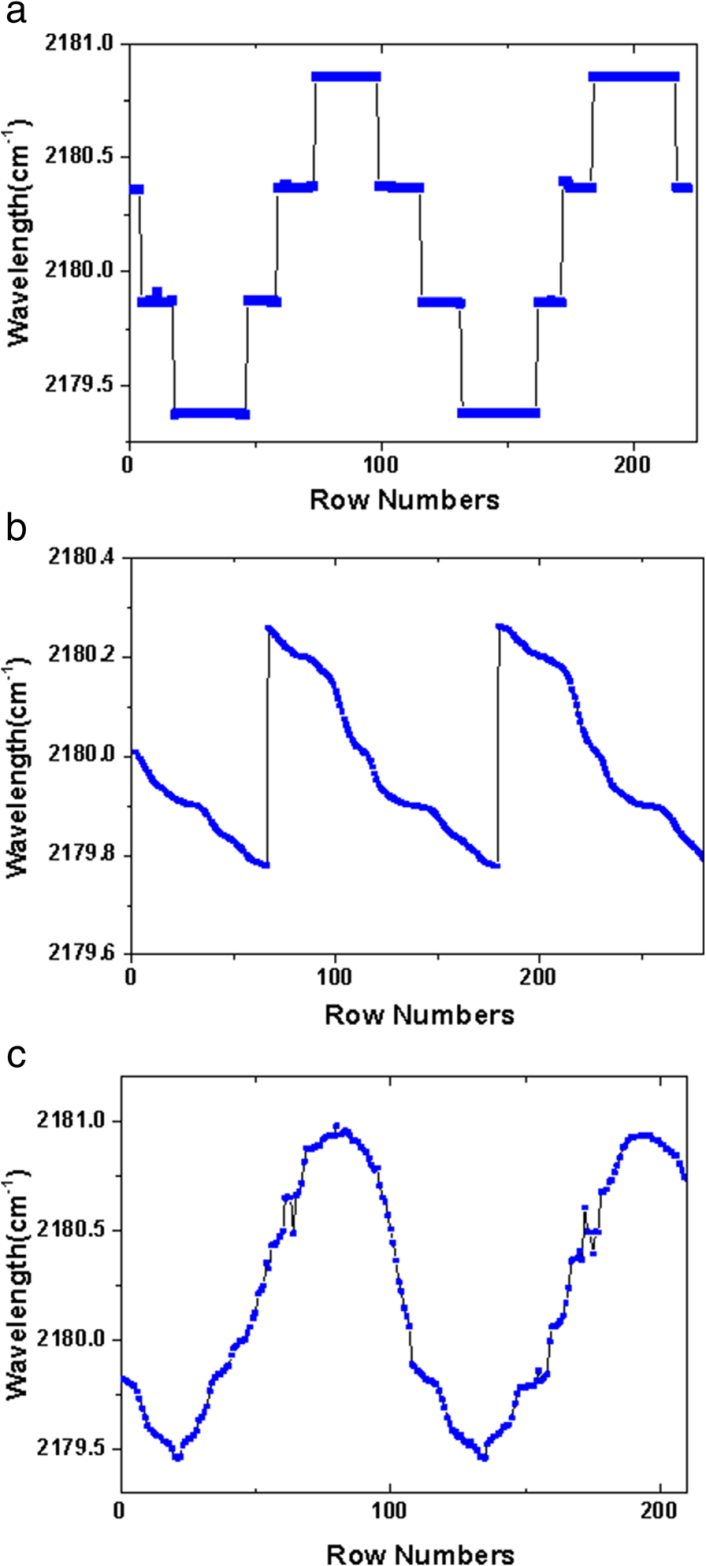
**a** The measured EC-QCL wavelength with the galvanometer voltage of 20 mV and the tuning frequency of 0.02 Hz. The mode hop is about 0.5 cm^−1^. **b** The measured EC-QCL wavelength tuning with a sawtooth wave modulation (0.02 Hz, 40 mA), which can compensate for the 0.5 cm^−1^ mode hop. **c** The measured EC-QCL wavelength with both galvanometer tuning and sawtooth wave modulation

We used a laser spectrum analyzer (Bristol Model 771) to evaluate the spectral resolution. Due to the minimum response time of about 0.5 s for the laser spectrum analyzer, we reduced the galvanometer frequency to 0.02 Hz which can record the complete wavelength tuning cycle. As shown in Fig. 5a, by changing the galvanometer angle, the wavelength varied discontinuously and mode hop about 0.5 cm^−1^ could be clearly identified. The mode hop is primarily associated with the FP modes of the QCL chip because of the non-ideal antireflection effect of the AR coating. In order to reduce mode hop spacing, we add a sawtooth wave modulation (0.02 Hz, 40 mA) to the DC driving current on the QCL chip with the galvanometer at a fixed angle. The wavelength tuning with the sawtooth wave modulation was shown in Fig. 5b. In one period, the wavelength is smoothly tuned to lower wavenumbers, which can compensate for the 0.5 cm^−1^ mode hop. However, it is noted that the wavelength tuning is not linear in one period, which is attributed to the temperature fluctuation of the QCL heat sink. The measured EC-QCL wavelength with both galvanometer tuning and sawtooth wave modulation was shown in Fig. 5c. Compared to Fig. 5a, the mode hop spacing has decreased to less than 0.2 cm^−1^.

## Conclusions

In summary, we have designed a fast swept-wavelength EC-QCL and investigated its performance, including single-mode selection, tuning range, and output power. The time-resolved step scan FTIR technique and laser spectrum analyzer were applied to measure the tuning range and spectral resolution. The EC-QCL could repeatedly swept at 100 Hz over its full tuning range of 135 cm^−1^ (about 290 nm) with a scan resolution of < 0.2 cm^−1^, which can be achieved with a sawtooth wave modulation. The CW threshold of the EC-QCL was as low as 250 mA with a maximum power of 20.8 mW. The low power consumption and fast swept-wavelength characteristic of the device could make it a promising light source for trace gas sensing applications.

## Abbreviations

AR: Anti-reflection
CW: Continuous-wave
DFB: Distributed feedback
EC-QCL: External cavity quantum cascade laser
FTIR: Fourier transform infrared spectrometer
HR: High reflectivity
MEMS: Micro-eletromechanical system
MIR: Mid-infrared
MOCVD: Metal-organic chemical vapor deposition
P-I-V: Power-current-voltage
QCLs: Quantum cascade lasers
SMSR: Side-mode-suppression-ratio
TEC: Thermoelectric cooler
